# Respiratory depression in a neonate born to mother on maximum dose sertraline: a case report

**DOI:** 10.1186/s13256-020-02640-1

**Published:** 2021-02-19

**Authors:** Greg J. Marchand, Katerina Meassick, Hannah Wolf, Sophia K. Hopewell, Katelyn Sainz, Sienna M. Anderson, Kelly Ware, Janelle Vallejo, Alexa King, Stacy Ruther, Giovanna Brazil, Kaitlynne Cieminski, Nicolas Calteux

**Affiliations:** 1 The Marchand Institute for Minimally Invasive Surgery, 10238 E. Hampton, Ste. 212, Mesa, AZ 85209 USA; 2 Midwestern University School of Osteopathic Medicine, Glendale Arizona, 85308 USA

**Keywords:** Sertraline, Neonatal exposure, Perinatal, Neonatal adjustment syndrome

## Abstract

**Background:**

Mood and anxiety disorders are common in women of childbearing age, especially during the peripartum period. As more women seek medical management for these conditions, there is an increasing need for studies to better examine the effects of exposure to selective serotonin reuptake inhibitors (SSRIs), and other antidepressants, on newborns at the time of delivery.

**Case presentation:**

We report the case of a term Caucasian infant born to a 17-year-old white female taking 100 mg of sertraline daily for depression and anxiety who exhibited respiratory depression and hypoxia after an uncomplicated vaginal delivery. The neonate was treated with the use of continuous positive airway pressure (CPAP) and supplemental oxygen and subsequently the symptoms resolved without complication.

**Conclusions:**

We present this case with the suspicion of poor neonatal adjustment syndrome as the possible cause of the respiratory depression and hypoxia in this newborn.

## Introduction

It is estimated that 15%–21% of peripartum women screen positive for perinatal mood disorders and that 14% of women of reproductive age (18–44 years old) screen positive for major or minor depression [1, 2]. In pregnant women who meet criteria for major or minor depression, lack of treatment has been associated with an increased risk of adverse outcomes for the infant, particularly during the later second or early third trimesters [3]. Current guidelines suggest antidepressant medication as the initial treatment of choice in both pregnant and postpartum patients. While the choice of antidepressant is based on a variety of criteria, SSRIS, specifically sertraline, are the mainstay of treatment as they are better studied and enjoy the support of more years of data. We report a case of transient hypoxia and perinatal depression in an infant born to a 17-year-old female who at the time of delivery was taking 100 mg of sertraline for symptoms of anxiety and depression.

## Case presentation

A 17-year-old white female gravida 1 para 0-0-0-0 with a past medical history of depression, panic attacks and right nephrolithiasis presented to a rural community hospital at 38 weeks 4 days for induction of labor. She had been receiving routine prenatal care. Patient’s pregnancy was complicated by right nephrolithiasis, young maternal age, a slipped disc in the lumbar spine and chlamydia during pregnancy. The decision was made to proceed with induction of labor at 38 weeks and 4 days rather than waiting for 39 weeks secondary to the patient’s nephrolithiasis and back pain from a slipped disc. This decision was made with the assistance of physicians who were board certified in maternal fetal medicine. Medications at the time of induction included sertraline 100 mg daily, macrobid 100 mg daily for urinary tract infections, and prenatal vitamins. She claimed compliance with these medications and denied any other medications, vitamins, or supplements. She had previously been prescribed escitalopram for her depression and anxiety but switched to sertraline 50 mg daily 4 months prior to delivery. Prior to this she had been on escitalopram for greater than two years. She felt that symptoms were not adequately controlled, so the dose of sertraline was later increased to 100 mg daily 2 months and 17 days prior to delivery. The last 100 mg dose of sertraline was given 5 h and 26 min prior to delivery. The patient denied use of tobacco, alcohol, or illicit drugs during pregnancy.

Patient was induced via 10 mg topical vaginal dinoprostone given one time and allowed to dilate. Subsequently she underwent an amniotomy after she was placed on oxytocin 20 unit in 1000 mL sodium chloride 0.9% infusion given at the rate of 1munit/min. She was allowed to progress through the normal stages of labor with no maternal or fetal complications. Continuous fetal monitoring was performed per hospital protocol, and tracing fluctuated between category I and category II without any unexpected decelerations or tracing abnormalities. At no time was cesarean section considered as fetal status appeared reassuring. There was no indication for biophysical profile during labor. Delivery occurred spontaneously. After delivery of the head it was noted that there was a loose nuchal cord which was easily slipped over the head of the infant and resolved. She gave birth vaginally to a 3373 g male infant at 0226 with APGAR scores of 3 at 1 min, 6 at 5 min and 8 at 10 min.

At the time of delivery, the newborn exhibited motor depression, cyanosis, and minimal respiratory effort. It was noted that the umbilical cord only had two vessels upon inspection. A pulse oximeter was placed at 2 min to monitor oxygenation and at four minutes it was noted that the infant was euglycemic with a blood sugar of 110 mg/dL. At nine minutes the infant continued to exhibit grunting, retractions, and tachypnea consistent with respiratory distress. He was found to have a respiratory rate of 30 and 82% oxygen saturation. He was then placed on 10 L of supplemental oxygen via simple bag mask. At ten minutes the APGAR score was 8, with points taken off due to cyanotic extremities and continued poor respiratory effort. The infant was then transferred to the neonatal intensive care unit (NICU) at 0240 for further evaluation and monitoring. In the NICU there was a sustained period of hypoglycemia which was initially noted at 0605 with a blood glucose of 42 mg/dL. Other than this, laboratory values and physical exam was normal for the duration of the hospitalization. The neonate remained on 2 L oxygen via nasal cannula until 1200 and was lowered to 1 L oxygen via nasal cannula which was continued for an additional 3.5 h. At this point the infant was weaned off supplemental oxygen and would not require it for the remainder of the hospitalization. The hypoglycemia persisted due to poor feeding with measurements of 63 mg/dL at 1202 and 68 mg/dL at 1803. At this point both the oxygen saturation and blood sugars both normalized and would continue to stay within normal limits until the patient discharged the following day at 1430. The patient was instructed to follow up with the pediatrician 2 days after discharge.

### Outcome

The pediatrician noted that there were no symptoms of respiratory distress or complications of the hypoxia in the newborn at 4 days postpartum.

## Discussion

As women of reproductive age are at an elevated risk for depression and anxiety, The American College of Obstetricians and Gynecologists issued a committee opinion in 2015 that recommends for the screening of depression both during pregnancy and postpartum [4]. This recommendation, as well as the changing attitudes towards mental health and its treatment, has led to an increase in women receiving treatment for peripartum depression and anxiety.

Current guidelines suggest antidepressant medication as the initial treatment of choice for peripartum depression, with psychotherapy as an acceptable alternative or adjuvant provided that the patient does not complain of suicidal ideation or significant cognitive impairment. The choice of antidepressant is informed by a number of considerations (tolerability, prior efficacy and use, potential fetal adverse effects, potential maternal adverse effects, use at conception, etc...). The most widely used class of medication has been selective serotonin reuptake inhibitors (SSRIs), with the exception of paroxetine [5]. About 80% of pregnant patients treated for depression in the first trimester receive SSRIs as opposed to other classes of antidepressants such as SSRIs, or monoamine reuptake inhibitors. Out of all the medications in pregnancy three SSRIs, sertraline, fluoxetine, and escitalopram, were among the twenty most commonly prescribed drugs in pregnancy, with sertraline being the drug of choice for treatment of anxiety and depression [6, 7]. While these three drugs have similar efficacy and a similar side effect profiles, they differ in their pharmacokinetic (PK) properties (Table 1) [8]. Many SSRIs have active metabolites, including sertraline and fluoxetine meaning that they may continue to exhibit effects until they are excreted. Metabolites are primarily excreted renally in urine, as well as through the gastrointestinal tract in fecal matter [9].

**Table 1:** Pharmacokinetics of three most prescribed SSRIs

Drug	Pharmaco-kinetics	Half life	Time to 99% metabolised [days]	Estimated exposure to parent drug [%]	Estimated exposure to metabolite [%]	Active metabolite [%]	Time to peak concentration [h]
Escitalopran1	Linear	27-33 h	6.1	73	70	No	3-4
Fluoxetine	Non-linear	1-4 days	25	58-73	63-71	Yes	6-8
Sertraline	Linear	26 h	5.4	29-73	29-63%	Yes	4-10

While numerous studies that demonstrate the lack of significant teratogenicity of SSRI use during pregnancy and confirm their safety during lactation, there are few to show the effects of their use at the time of or immediately prior to the time of delivery [10]. Studies have shown that exposure to SSRIs during pregnancy is associated with increased infant morbidity which necessitates admission to the NICU as well as a collection of transient symptoms referred to as poor neonatal adjustment syndrome (PNAS) [11]. This syndrome is characterized by, but not limited to, respiratory distress, hypoxia, seizures, and limpness (Table 2) [12]. Early clinical and research findings of this syndrome led to a 2004 warning by The United States Food and Drug Administration recommending that SSRIs be tapered 7–10 days prior to delivery [13]. Currently, the tapering of SSRIs prior to delivery is not recommended as there is insufficient evidence to suggest that doing so is beneficial [14–16]. While the pathophysiology of PNAS is not fully understood, these symptoms appear similar to those seen in adults overdosing or withdrawing from SSRIs. There are few studies that have assessed the safety during the third trimester, but current literature suggests that there is an increased risk of perinatal complications including respiratory distress, irritability and feeding problems [17]. Additionally, in a study of 700,000 infants with antenatal exposure to SSRIs, when controlled for all other factors, were more likely to manifest central nervous related symptoms, respiratory distress, hypoglycemia, and persistent pulmonary hypertension [3]. From studies looking at fetal cord blood and amniotic fluid versus maternal plasma concentrations of SSRIs we know that varying concentrations of the parent drug and metabolites of SSRIs pass through the placenta (Table 1). For sertraline, the estimated exposure of 29–73% of the parent drug and 29–63% of the active metabolite [18].

**Table 2:** Symptoms of Poor Neonatal Adjustment Syndrome, Selective Serotonin Reuptake Inhibitor Overdose and Selective Serotonin Reuptake Inhibitor withdrawal

PNAS	SSRI overdose	SSRI withdrawal
Autonomic Instability Diaphoresis^a^^,b^ Exaggerated Moro Reflex Feeding difficulties Fever^a^ Gastrointestinal Disturbance^a,b^ Hypoglycaemia Increased Muscle Tone^a^ Insomnia^a^ Irritability^a^^,b^ Limpness Persistent Crying Poor thermoregulation Respiratory Distress Seizures^a^ Sleep Disturbance^b^ Tachycardia^a^ Tachypnoea^b^ Tremors^b^	Agitation Anythmia Clonus Confusion Diaphoresis^a^ Fever^a^ Gastrointestinal disturbance^a^ Headache Hyperreflexia Hypertension Increased Muscle Tone^a^ Insomnia^a^ Irritability^a^ Loss of consciousness Loss of muscle coordination Mydriasis Piloerection Seizures^a^ Shivering Tachycardia^a^ Tachypnoea Twitching	Agitation Ataxia Diaphoresis^b^ Dizziness Gastrointestinal Upset^b^ Headache Insomnia^b^ Irritability^b^ Lethargy Nausea Paraesthesia Sleep disturbance^b^ Tremor^b^

^a^Overlapping symptoms between PNAS and SSRI overdose

^b^Overlapping symptoms between PNAS and SSRI withdrawal

Few studies have assessed the effects at the time of delivery, but research consistently shows the use of SSRIs during pregnancy are related to a variety of neonatal complications including respiratory distress. We were able to find no specific studies addressing neonatal symptoms that were thought to be from maternal ingestion of sertraline prior to delivery, other than those addressing withdrawal from sertraline. Further research is additionally needed to elucidate whether these complications represent a direct serotonergic effect on an immature nervous system or are a product of drug overdose or withdrawal. Many of the symptoms of an SSRI overdose and withdrawal overlap with those of PNAS (Table 2). Symptoms such as gastrointestinal upset, tremors, sleep disturbances, tremors or twitching are seen in all three, while symptoms unique to PNAS include hypoglycemia, respiratory distress, and tachypnea [19, 20]. In order to have symptoms of withdrawal, discontinuation of the drug would need to occur with sufficient time prior to delivery for the active component to be fully metabolized or excreted from both maternal and fetal circulation. It is currently not well understood how the timeline of overdose or withdrawal is altered by transport across the placenta or by altered levels of fetal metabolism. The time required for >99.00% of a drug to be excreted depends on the half-life and pharmacokinetics of the drug, ranging from 5.4 days with sertraline up to 25 days with fluoxetine (Table 1). Therapeutic doses of SSRIs are based on an adult cytochrome P450 mediated metabolism, this system is not fully functioning and develops at different rates during the postnatal period. While many of these cytochromes develop rapidly after birth, at the time of birth they are not fully functional. This window of time between birth and full development of a cytochrome P450 (CYP450) system could potentially lead to reduced metabolism and increased concentrations in the neonate. The cytochrome primarily responsible for metabolism of SSRIs is CYP2D6, as well as CYP3A4. While CYP2D6 starts at low levels in the fetus and rapidly develops in the first month postpartum, the CYP3A system has a transition from CYP3A7 to CYP3A4 which reaches 30–50% at 3–12 months of age [21]. Metabolites from the cytochrome P450 system are then excreted renally in the urine, as well as by the gastrointestinal tract in fecal matter. While nephrons are structurally complete by 36 weeks gestation, the newborn kidney is still functionally immature. During the first weeks of life renal function undergoes a rapid maturation, reaching a mature glomerular filtration rate corrected for body size by 12 months of age [22]. During pregnancy, there are increased rates of maternal cytochrome P450 expression and activity and during labor there continues to be transplacental blood exchange allowing for adequate metabolism and excretion of the drug. But after delivery, there is no longer blood exchange between the mother and infant, leaving any parent drug or metabolite in the infant’s blood. This leaves a period where the infant has both reduced levels of cytochrome activity and kidney function, creating a window of vulnerability to the drugs' effects.

In this case we see that the patient had been treated with sertraline, the last dose of which was given 5 h and 26 min prior to delivery. This time is sufficient to allow for peak concentration of the drug to be reached in maternal circulation and move through the placenta into fetal circulation and amniotic fluid. Without fully functional mechanisms to metabolize the parent drug or excrete the active metabolite, a window of vulnerability was present in the neonate to the effects of sertraline. Research currently demonstrates the link between the use of SSRIs during pregnancy and PNAS. Despite not fully understanding the pathophysiology it cannot be ignored that the symptoms of PNAS overlap with those known to be caused by an overdose of SSRIs. In this case the neonate presented with transient respiratory depression, hypoxia, feeding difficulties and hypoglycemia, all of which are consistent with a diagnosis of PNAS. Without other clear etiologies for these symptoms, it is reasonable to conclude that they can be linked to the use of high dose sertraline at the time of delivery.

## Conclusions

Mood and anxiety disorders are common in women of childbearing age and there are increased rates in those during the peripartum period. As treatment with antidepressant medications, particularly SSRIs, is widely accepted as the first line treatment in pregnant women it is paramount that we fill the current gap in knowledge of the consequences of exposure to SSRIs and other antidepressants on newborns at the time of delivery. These medications are some of the most commonly prescribed drugs during pregnancy, consequently there is a need for further studies to evaluate the potential effects and risks of usage.

## Abbreviations

APGAR: Appearance, Pulse, Grimace, Activity and Respiration
NICU: Neonatal intensive care unit
SSRI: Selective serotonin reuptake inhibitor
PNAS: Poor neonatal adjustment syndrome
CYP450: Cytochrome p450
